# Biogeography of photoautotrophs in the high polar biome

**DOI:** 10.3389/fpls.2015.00692

**Published:** 2015-09-11

**Authors:** Stephen B. Pointing, Burkhard Büdel, Peter Convey, Len N. Gillman, Christian Körner, Sebastian Leuzinger, Warwick F. Vincent

**Affiliations:** ^1^Institute for Applied Ecology New Zealand, School of Applied Sciences, Auckland University of Technology, Auckland, New Zealand; ^2^Department of Biology, University of Kaiserslautern, Kaiserslautern, Germany; ^3^British Antarctic Survey, NERC, Cambridge, UK; ^4^National Antarctic Research Centre, University of Malaya, Kuala Lumpur, Malaysia; ^5^Institute of Botany, University of Basel, Basel, Switzerland; ^6^Centre d’\Études Nordiques and Département de Biologie, Université Laval, Québec, QC, Canada

**Keywords:** antarctic, arctic, bryophytes, cryptogams, cyanobacteria, lichen, plant biogeography

## Abstract

The global latitudinal gradient in biodiversity weakens in the high polar biome and so an alternative explanation for distribution of Arctic and Antarctic photoautotrophs is required. Here we identify how temporal, microclimate and evolutionary drivers of biogeography are important, rather than the macroclimate features that drive plant diversity patterns elsewhere. High polar ecosystems are biologically unique, with a more central role for bryophytes, lichens and microbial photoautotrophs over that of vascular plants. Constraints on vascular plants arise mainly due to stature and ontogenetic barriers. Conversely non-vascular plant and microbial photoautotroph distribution is correlated with favorable microclimates and the capacity for poikilohydric dormancy. Contemporary distribution also depends on evolutionary history, with adaptive and dispersal traits as well as legacy influencing biogeography. We highlight the relevance of these findings to predicting future impacts on diversity of polar photoautotrophs and to the current status of plants in Arctic and Antarctic conservation policy frameworks.

## Introduction

The polar regions exhibit a polar frost climate (Peel and Finlayson, 2007) with summer mean temperatures near freezing. Historically, however, they were significantly warmer and plant paleo-biogeography reflects this. Extensive rainforests occurred in both polar regions at least from the Cretaceous until the Miocene, when significant global cooling occurred (Herman and Spicer, 2010; Cantrill and Poole, 2012). Post-glacial recolonisation then occurred from multiple refugia that were abundant at least in the Arctic (Abbott and Brochmann, 2003). A striking feature of contemporary polar landscapes is that high-stature vascular plants (trees and shrubs > 0.5 m tall) are largely absent, and completely so from the Antarctic. This reflects a broader latitudinal transition in plant composition from tree-dominated landscapes in non-arid temperate and tropical latitudes, to a sub-polar tundra dominated by shrubs and dwarf trees, and finally beyond the limit for shrubs to High Arctic and Antarctic regions where vascular plant life is relatively or extremely restricted, respectively. This structural trend parallels the negative latitudinal gradient in productivity and species richness (Gillman and Wright, 2010; Gillman et al., 2014). Contemporary polar diversity also displays a clear dichotomy, with the Arctic supporting thousands of extant vascular plant species (Walker et al., 2005) whereas the Antarctic has only two, and these are restricted to the Antarctic Peninsula (Cantrill and Poole, 2012). Here we illustrate contemporary biogeographic patterns for vascular plants, non-vascular plants and lichenised and free-living photoautotrophic microorganisms; herein collectively referred to as plants to reflect the importance of microbial photoautotrophy in polar regions (Aleksandrova, 1988; Vincent, 2000; Jungblut et al., 2010). We review their historical biogeography and present an explanation for major transitions in contemporary communities. We identify how microclimate and organismal traits drive this biogeography and explain how the disparity in plant distribution between the Arctic and Antarctic is a result of temporal and dispersal barriers. Finally, we evaluate conservation status for polar plants within the context of current Arctic and Antarctic treaties and policy frameworks.

## The High Polar Biome

**FIGURE 1:**
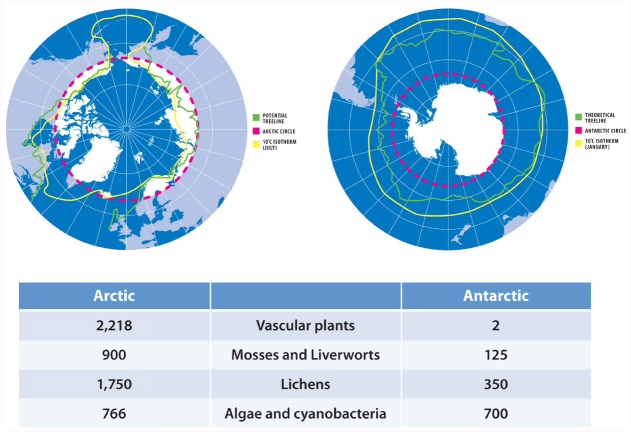
**Extent of contemporary Arctic and Antarctic habitats for polar photoautotrophs.** Red line: Arctic/Antarctic Circle; Yellow line: 10°C summer isotherm; Green line: treeline. Arctic treeline calculated as extent of summer mean temperature at or above 6.4°C, with the growing season defined as the sum of days with a daily mean temperature of 0.9°C and not falling below 94 such days (blue line; Paulsen and Körner, 2014). Biodiversity data shows number of species, and was collated from the National Snow and Ice Data Center (https://nsidc.org/cryosphere/frozenground/plants.html) and Arctic Biodiversity Assessment (http://www.arcticbiodiversity.is/the-report/chapters/plants) (Arctic), and British Antarctic Survey (http://www.antarctica.ac.uk/about_antarctica/wildlife/plants) and Australian Antarctic Division (http://www.antarctica.gov.au/about-antarctica/wildlife/plants) (Antarctic).

The highest latitudes on Earth are permanently cold due to their obliquity toward the sun as a result of the planet’s rotational tilt. These polar regions comprise the Arctic in the north and the Antarctic in the south, defined by circles of latitude at approximately 66.5° that mark the limit where the Sun can remain continually above or below the horizon throughout a 24 h period (Marsh and Kaufman, 2012). Polar regions have also been delineated using the treeline and the 10°C summer isotherm (UNEP, 2010). Such criteria are not static, since they vary with the Earth’s tilt and climate, while treeline can be misleading in that trees may be absent from otherwise favorable sites because of local circumstances, including microclimate and disturbances (Körner, 2012c). The terrestrial Arctic comprises approximately 11,000,000 km^2^ of landmass from eight nations, much of which is contiguous with temperate landmass at lower latitudes (UNEP, 2010; Figure 1). Conversely, the terrestrial Antarctic includes approximately 14,000,000 km^2^ of mostly permanently ice-covered landmass and is a large isolated Antarctic continent managed by an international treaty, plus small outlying sub-Antarctic islands governed by five sovereign nations (UNEP, 2010; Figure 1).

Polar regions support significant amounts of seasonally or permanently ice-free land, which although relatively oligotrophic (Hugelius et al., 2014) is colonized by photoautotrophs. The Arctic sub-polar and polar tundra areas are seasonally ice-free, and a small percentage of high latitude polar land is permanently ice-free polar desert due to low precipitation and sublimative loss of snowfall (e.g., 45,000 km^2^ in Antarctica, approximately 0.32% of terrestrial surface, Moorhead et al., 1999). Arctic temperatures generally range from –40 to 0°C in winter to –10 to 0°C in summer, although radiative effects on exposed rocky ground can raise temperatures as high as 30°C in some locations (NSIDC, 2014). Precipitation as rain and snow varies by an order of magnitude across the Arctic but can be very low. For example, in the polar desert of the Arctic Basin, mean annual precipitation is approximately 250 mm/y (NSIDC, 2014). The Antarctic is colder than the Arctic, and holds the record for the coldest recorded temperature on Earth (–93.2°C during 2013) on the ice sheet of the central plateau (Turner et al., 2013). Areas that experience ice-free periods are considerably warmer. For example, the west Antarctic Peninsula experiences low positive mean air temperatures for up to 3–4 months of the year and precipitation of up to 500 mm/y (Turner et al., 2005). Conversely, the McMurdo Station in East Antarctica (close to Antarctica’s largest permanently ice-free area, the McMurdo Dry Valleys) experiences mean temperatures of –5.5 to –0.2°C in summer and –30.1 to –21.7°C in winter (Turner et al., 2005). Precipitation levels in the McMurdo Dry Valleys are lower than 50 mm annually, occurring entirely as snow (Turner et al., 2005).

## Historical Biogeography of Polar Photoautotrophs

**FIGURE 2:**
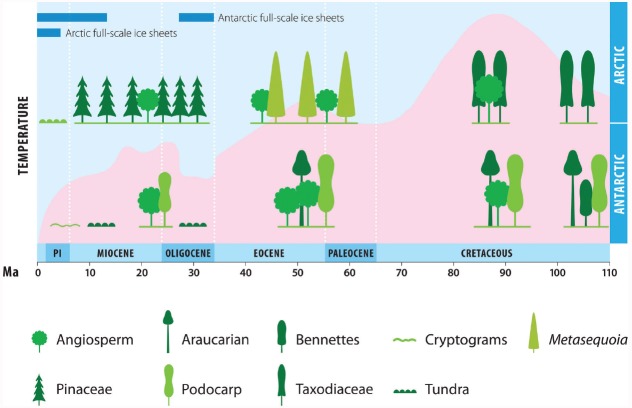
**Historical biogeography of polar photoautotrophs.** Historic climate and vascular plant biodiversity in the Arctic and Antarctic. Pink represents Southern Ocean ice-free sea-surface relative temperature. Cooling in the Arctic was less severe during the Neogene than in Antarctica (Huber, 1998; Zachos et al., 2001). Plant symbols reflect general morphology of each group and are not to scale. Pl indicates Pliocene.

Antarctica rafted over the pole during the early Cretaceous supporting productive rainforests with trees 40m high (Cantrill and Poole, 2012). By the mid Cretaceous deciduous taxodiaceae forest also occurred in the Arctic up to 85°N. Angiosperms migrated into high latitudes of both poles ∼15 My after appearing at lower latitudes (Herman and Spicer, 2010; Cantrill and Poole, 2012). By the Cretaceous thermal maximum, the global temperature gradient was almost flat (0.10 vs. 0.40°C/degree latitude today; Huber et al., 2002; Hay and Floegel, 2012) and diverse forest dominated by angiosperms occurred at both poles (Spicer and Herman, 2010; Cantrill and Poole, 2012; Figure 2). Global cooling ensued during the late Cretaceous in the Arctic and Antarctic (Moran et al., 2006) causing shifts toward cooler temperate forests (Cantrill and Poole, 2012; Falcon-Lang et al., 2004) that nonetheless remained productive (Williams et al., 2009; Spicer and Herman, 2010) and free of mass extinctions across the Cretaceous–Paleogene boundary (Herman et al., 2004; Spicer and Herman, 2010; Cantrill and Poole, 2012).

Temperatures peaked again in the Eocene (50–52 Ma) and by the middle-late Eocene there is evidence of diverse *Nothofagus*-podocarp forest in Antarctica (Francis, 2013) and diverse *Metasequoia* forests up to 40 m tall in the Arctic (Williams et al., 2009; Eberle and Greenwood, 2012; Harrington et al., 2012; Figure 2). With the sharp decline in temperatures at the Eocene-Oligocene transition, diversity fell at both poles. The Arctic conifer–broadleaf forests with frost-sensitive taxa such as palms were displaced by less-diverse Pinaceae-dominated forests (Eldrett et al., 2009). In Antarctica forests were replaced by fellfield or tundra vegetation (Truswell and Macphail, 2009), a trend that was temporally reversed in the warm early Miocene when woody stature returned (Cantrill and Poole, 2012).

Further cooling in the late Miocene led to cold boreal forests replacing temperate forests in the Arctic (Pound et al., 2012; Figure 2). In Antarctica, cooling was more severe and forests were again replaced by fellfield. Stature and diversity in Antarctica continued to decline until by the early Quaternary all vascular plants were probably lost (Cantrill and Poole, 2012). Less severe cooling in the Arctic (Ballantyne et al., 2010; Brigham-Grette et al., 2013) led to a circum-Arctic belt of tundra replacing forests by 3 Ma (Abbott and Brochmann, 2003). When Earth entered the Pleistocene ice-house, glaciated Antarctica remained isolated by the Southern Ocean, whereas extensive non-glacial refugia are thought to have extended beyond the Arctic Circle and continental refugia remained to the south of the ice sheets (Abbott and Brochmann, 2003). As the climate warmed in the Holocene, pre-existing Arctic tundra, dominated by flowering herbs, was replaced by one with a far greater abundance of grasses and woody vegetation (Willerslev et al., 2014), whereas only two vascular species occur naturally in Antarctica (Cantrill and Poole, 2012), both extending to a southern limit of c. 69°S on northern Alexander Island (Convey et al., 2011).

## Contemporary Biogeography of Polar Photoautotrophs

A major delineation for contemporary plant biogeography is the treeline, here defined as the natural absence of trees (Figure 1). The low temperature treeline may range substantially beyond the Arctic Circle, reaching latitudes as far north as 72°on the Taymyr Peninsula. Beyond the Arctic treeline, heathland occurs composed of dwarf shrubs and graminoids such as sedges and rushes of the Cyperaceae and Juncaceae (Daniels et al., 2013). Small patches of these reach high latitudes, with more than 20 angiosperm species described for the northern edge of Greenland (Bay, 1992) and 64 described for the northern edge of Canada (Vincent et al., 2011). The Antarctic does not have a treeline, because this vegetation boundary is located at lower latitudes than the Antarctic Circle (Figure 1). Although continental Antarctica is generally colder than the Arctic, this is not true for the Antarctic Peninsula, which features comparable climatic conditions to northern Greenland but only hosts two vascular plant species; the Antarctic hair grass (*Deschampsia Antarctica*) and Antarctic pearlwort *Colobanthus quitensis*, (Cantrill and Poole, 2012). Based on climatic data, however, the Antarctic should host more vascular plant life than is currently present.

Beyond the latitudinal limit of angiosperms, plant life is restricted to non-vascular cryptogams. Here the bryophytes become a major plant cover, with the Arctic supporting hundreds of described mosses and liverworts covering about half the Arctic (Walker et al., 2005), whereas the Antarctic has only approximately 100 described moss and liverwort species that cover a small fraction of Antarctica’s total land area (Seppelt and Green, 1998; Figure 1). A diversity of species occur in soil and submerged habitats in the Arctic tundra (Daniels et al., 2013), with relatively fewer species, commonly *Bryum* and other genera in higher latitude polar desert locations (Seppelt and Green, 1998; Daniels et al., 2013). Because water is a limiting factor for productivity such patterns are consistent with the predominantly linear relationships found between species richness and productivity (Gillman et al., 2015). Bryophytes may, however, be locally abundant and form extensive moss beds even at high latitudes where sufficient moisture occurs, for example near ephemeral runoff streams from glacier meltwater.

The largest and most complex terrestrial biocoenoses of polar regions are biological soil crusts (Belnap et al., 2003). These comprise high species richness but very few (2–3) trophic levels supported by cyanobacterial and chlorophyte photautotrophs, plus fungi, lichens and bryophytes in different proportions (Figure 1). A recent assessment of one Antarctic biological soil crust revealed it supported 66 cyanobacteria, 44 algae, 42 lichens, and 14 bryophyte species (Büdel and Colesie, 2014). Extreme polar desert soils such as those of the McMurdo Dry Valleys of Antarctica and the Arctic Basin support relatively less soil crust cover, although prolific growth of cyanobacterial mat (dominated by Nostocales and Oscillatoriales) occurs in lakes and streams (Bonilla et al., 2005; Taton et al., 2006).

Under the most extreme conditions photoautotrophic life is restricted to unicellular taxa (Chlorophyta and Cyanobacteria) occurring as cryptic biofilms or in lichen symbioses on the surface (epilithic), within (endolithic), or beneath (hypolithic) exposed rock substrates (Cockell and Stokes, 2004; Pointing et al., 2009). Nonetheless, the extent of this standing photosynthetic biomass is appreciable even at the most pole-ward locations for ice-free land (Pointing et al., 2009). These rock-inhabiting photoautotrophs are restricted to relatively few cyanobacterial genera e.g., *Chroococcidiopsis*, *Leptolyngbya*, *Nostoc*, *Phormidium*, (Friedmann, 1982; De La Torre et al., 2003; Cockell and Stokes, 2004; Wood et al., 2008; Pointing et al., 2009; Yung et al., 2014) and the chlorophyte lichen phycobiont *Trebouxia* (Yung et al., 2014). Lithic colonization appears to have no latitudinal extinction limit. Chlorophyte algae also seasonally colonize snowpack and sea ice (Muller et al., 2001; Fujii et al., 2010).

## Drivers of Biogeography for Polar Photoautotrophs

**FIGURE 3:**
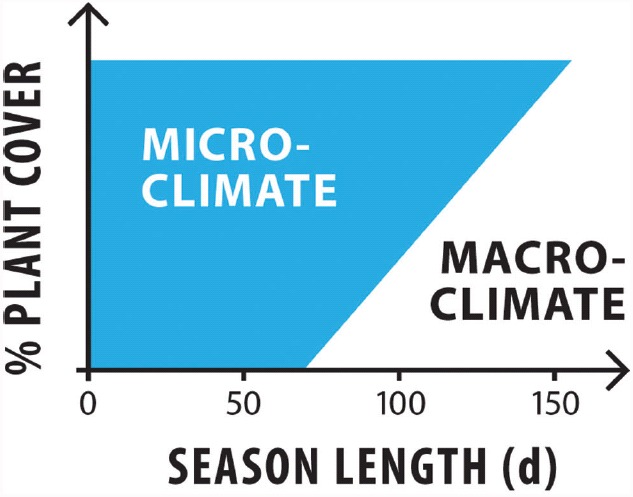
**Relative role of microclimate and macroclimate as abiotic drivers for polar photoautotrophs.** Areas with a relatively short growing season show from 0 to 100% plant cover (Paulsen and Körner, 2014), and macroclimate assumes a greater role as season length increases. For example with a season length of c. 150 days, the plant cover is mainly driven by macroclimate. As season length decreases, an increasing proportion of the total plant cover is driven by microclimate, such that at a season length of 70 days or less, plants exclusively rely on microclimatic effects, which are decoupled from macroclimate.

Low temperature and water availability ultimately limit all plant life on Earth. However, rather than the mere absence of water or the prevalence of low temperatures, we argue that it is the temporal dynamics of environmental conditions that are critical for presence/absence thresholds of photoautotrophs. There is no ice-free land on Earth with a climate that prevents plant life permanently, i.e., with moisture-free soils or surface temperatures consistently < 0°C (Peel and Finlayson, 2007). The necessary cellular processes to sustain plant life (photosynthesis, respiration, cell growth and division) are at least during certain periods, possible anywhere. Soils are relatively oligotrophic in polar latitudes but are not a barrier to plant colonization or to establishment of mycorrhizae (Oehl and Körner, 2014). We propose that the limiting factor for plants is instead related to their exposure to the surrounding climate, and this is illustrated in Figure 3. Given the right microclimate, almost any area in the Arctic could develop a 100% plant cover, regardless of the theoretical season length. This is illustrated in Figure 3: A season length of c. 150 days, the plant cover is mainly driven by macroclimate. As season length decreases, an increasing proportion of the total plant cover is driven by microclimate, such that at a season length of 70 days or less, plants exclusively rely on microclimatic effects, which are decoupled from macroclimate.

Here, we discuss the drivers of transitions between four major functional groups of plants: We distinguish between trees (woody, erect plants, including tall shrubs), herbaceous plants (low-stature plants including prostrate shrubs), cryptogams (non-seed multicellular plants, including bryophytes, lichens, and biological soil crusts), and microbial photoautotrophs (chlorophyte and cyanobacterial biofilms in soil and rock).

### From Trees to Herbaceous Plants

**FIGURE 4:**
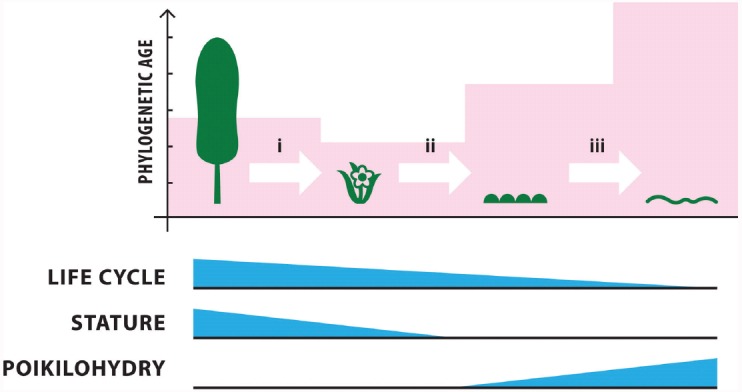
**Key biotic drivers for transition between polar photoautotrophic groups.** The relative influence of life cycle, stature and poikilohydry are shown by the extent of blue triangles (taller shading = greater influence). (i) Macroclimatic conditions largely drive the transition from trees to low stature shrubs via aerodynamic coupling of the tall stature of trees. Whilst trees are exposed to ambient air temperature, shrubs can more easily decouple from atmospheric conditions due to their low stature (Körner, 2012a). (ii) The transition from low stature shrubs to cryptogams is driven by via ontogeny barriers (Pannewitz et al., 2003; Green et al., 2011; Körner, 2011). (iii) Microclimate drives the presence/absence of most higher plants through exposure (radiative heat) and wind, whilst water mostly drives the transition from higher cryptogams (mosses) to highly poikilohydric unicellular plants (cyanobacteria, Potts, 1999). Phylogenetic age correlates well with tolerance of extreme conditions and relative phylogenetic age is shown by the pink shading (taller shading = older lineage).

One of the factors that has contributed to the success of trees around the globe is their tall stature, yet this becomes a disadvantage in polar regions (Figure 4). This is due to stature creating an intimate coupling to air temperature. Infrared thermography illustrates that trees cannot decouple aerodynamically from prevailing low air temperatures as low stature vegetation does (Körner, 2012b). Tree foliage will rarely warm more than 5°C above air temperature (Leuzinger and Körner, 2007). The Arctic treeline is thus a phenomenon mainly driven by macroclimate, much like the alpine or Arctic snowline (Körner, 2012a). For both the alpine and Arctic environments the treeline can be described as a line where the summer mean temperature reaches 6.4°C, with the growing season defined as the sum of days with a daily mean temperature of 0.9°C and not falling below 94 such days (Paulsen and Körner, 2014; Figure 1). The predictive power of this temperature-based delineation of the Arctic and alpine treeline is high and matches modeling attempts that account for biological thresholds of tissue formation (i.e., sink limitation, Leuzinger et al., 2013) as obtained for instance for xylogenesis in trees (Lenz et al., 2013) or leaf expansion in winter crops (Körner, 2008). Apart from adequate air temperatures, higher stature plants require sufficient non-frozen ground for root growth and moisture/nutrient uptake, and thus permafrost depth can also be a limiting factor (Körner and Hoch, 2006). Cold temperatures never exert photosynthetic C-fixation limitations since the theoretical lower thermal limit for plastid-mediated photosynthesis occurs where chloroplasts freeze at –5 to –8°C (Körner, 2003a) which is beyond the limit for moisture availability. Cold adapted photosynthetic tissue in vascular plants reaches 60–70% of maximum rates at 5°C and 30–40% at 0°C, when growth is 0 (Körner, 2003b).

In contrast to trees, lower stature herbaceous plants with more shallow root systems can exploit favorable soil microclimates. For example, at latitudes as high as 78°N in Svalbard, low stature Arctic tundra can warm up under 24 h sunlight in summer to temperatures above 20°C (Scherrer and Körner, 2009), which is very similar to the temperatures encountered at lower alpine latitudes. Thermal mapping of near-surface soils over a full season indicates seasonal mean temperatures at 2–3 cm soil depth where many herbaceous plants keep their apical meristems are consistently 2–4°C above 2 m air temperature, with mosaics of microhabitats exhibiting seasonal means as warm as 8°C above air temperature (Scherrer and Körner, 2009; Larcher, 2012). Hence, these low stature plants exploit a microclimate that deviates substantially from atmospheric conditions (Figure 3). This ability to decouple from air temperature permits low stature plant life near the ground to reach high latitudes of up to 84°N and elsewhere at elevations as high as 6400 m in the Himalayas at 30°N (Körner, 2011).

### From Herbaceous Plants to Cryptogams

**FIGURE 5:**
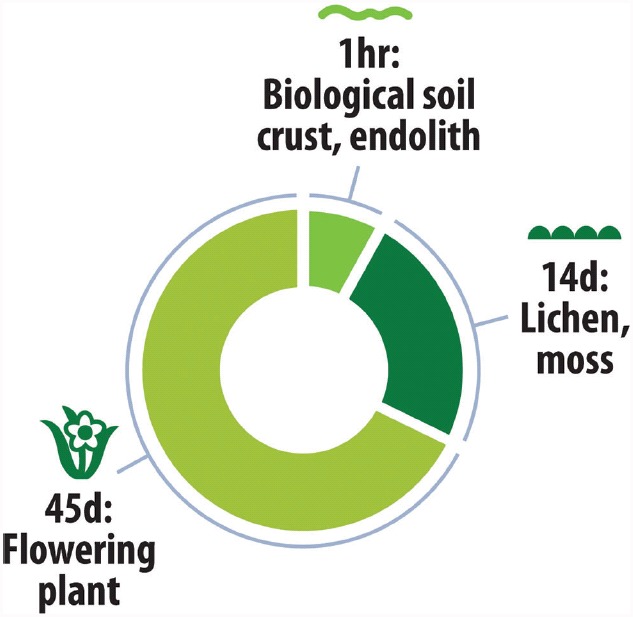
**Minimum favorable microclimate period for completion of life cycle by polar photoautotrophs.** Vascular plants require a minimum of 45 days with temperatures that permit growth (Körner, 2011). In contrast cryptogamic bryophytes and lichens likely require only between 10 and 14 days to achieve net positive carbon balance (Pannewitz et al., 2003; Green et al., 2011) and for unicellular photoautotrophs this may be 1 h or less (Potts, 1994).

At the poles seed plants become victims of their own success, whilst the evolution of seeds and water conducting tissue contributes much to the competitive advantage of plants at warmer latitudes, the cost of their production is a serious limitation to plant life in the cold (Figure 4). The specific challenges of seed plants arise from the complexity of their seasonal life cycle, tissue composition and plant architecture. Here ontogeny can be a major limitation because for an angiosperm leaf to develop and mature, redeem its own carbon costs and supply roots, stems and flowers with assimilates, amortization requires a minimum of 45 days with temperatures that permit growth functioning (Körner, 2011). In contrast cryptogamic bryophytes that seldom produce sporophytes in polar regions (Ochyra et al., 2008) and lichens probably require only between 10 and 14 days to achieve net positive carbon balance (Pannewitz et al., 2003; Green et al., 2011) and so can exploit shorter growth periods (Figure 5).

There are also thermodynamic constraints for plants imposed by macroclimate. Optimal growth temperatures recorded for polar vascular plants may be as high as 14°C (Xiong et al., 1999) and up to 23°C for bryophytes (Uchida et al., 2002). Vascular plants require a minimum of 5°C for reasonable rates of tissue growth and differentiation (Körner, 2008), although very slow cell production may occur close to 0°C (Larcher, 2012). One explanation as to why growth of stems and foliage in angiosperms is inhibited at such temperatures whilst cryptogams and unicellular plants survive may be related to lignin synthesis in xylem. This is one of the few biochemical processes that is not enzyme driven, but relies on temperature-dependent auto-polymerization of monomers. Another reason may be the formation of a thick cellulose secondary cell wall in angiosperms, compared to the relatively thin cell wall in monadic green algae or cyanobacteria.

The second major disadvantage of the otherwise highly efficient xylem architecture of flowering plants is its inability to survive complete desiccation. Polar environments exhibit matric (air drying) stress that causes severe hypertonicity and eventually desiccation in plants. The adverse cellular effects on protein assembly, gene expression and membrane integrity require that a mechanism to ensure controlled anhydrobiosis is achieved without loss of viability (Billi and Potts, 2002). Bryophytes are multicellular plants and can therefore partition water storage from photosynthetic and other functions in their thallus. Under severe xeric stress plants undergo desiccation, and this appears to be a constitutive ability. For example bryophytes are consistently recorded with high levels of compatible solutes in their cells (Green et al., 2011). Non-vascular plants are poikilohydric; they possess the essential ability to survive in a dormant state during desiccation and then resume biological activity upon re-wetting (Potts, 1994; Wasley et al., 2006; Kranner et al., 2008; Charron and Quatrano, 2009). Desiccation tolerance appears to be a plesiotypic adaptation in cryptogams in that it is not specific to polar taxa and indicates that these phyla are “pre-adapted” to polar colonization. Mosses are generally more capable of tolerating prolonged desiccation than lichens, although their re-activation may be a slower process than in lichens (Green et al., 2011). Such traits are precluded in vascular plants due to their relatively high degree of tissue and life cycle specialization (Körner, 2003a). The ability to respond rapidly enough to exploit transient conditions of moisture sufficiency and to withstand prolonged periods of moisture deficit contributes to the enduring success of cryptogams in the polar biome (Convey, 1996).

### From Cryptogams to Microbial Photoautotrophs

In the most extreme high polar environments, a final transition occurs from multicellular cryptogams to unicellular algae and cyanobacteria. Even during short windows of favorable microclimatic conditions the cyanobacteria thrive at low temperatures (Vincent, 2000; Pointing et al., 2009; Bahl et al., 2011) with virtually no minimum time required for these physiological temperatures to be present, which allows them to opportunistically “live by the hour” (Convey, 1996). A key advantage for lichenised and free-living chlorophytes and cyanobacteria is that their cytology and biochemistry permit photoautotrophic metabolism at temperatures close to 0 (Kappen and Friedmann, 1983; Tang and Vincent, 1999), and even the capacity of some species to gain positive net photosynthesis at sub-0 temperatures, for example as low as –18.5°C for the Antarctic lichen *Neuropogon acromelanus* (Lange and Kappen, 2013) in the laboratory and similar values reported in the field for *Usnea sphacelata* and *Umbilicaria aprina* (Kappen, 1989; Schroeter et al., 1994).

The cyanobacterial response to desiccation involves secretion of intracellular compatible solutes such as trehalose and sucrose (Potts, 1999; Billi and Potts, 2002; Kranner et al., 2008). Polar cyanobacteria invest considerable resources into secretion of an extracellular polymeric substance rich in polysaccharides and other protective substances, and this has been implicated in desiccation and other stress tolerance (Knowles and Castenholz, 2008). They also possess a wide array of osmotic stress tolerance genes, as revealed by a recent metagenomic study of Antarctic cyanobacteria (Chan et al., 2013). Other cellular protective functions such as DNA repair mechanisms and secretion of chaperone proteins are likely important, as demonstrated for the extreme radiation and desiccation tolerant bacterium *Deinococcus radiodurans* (Cox and Battista, 2005). Due to their simple cellular architecture and clonal growth, cyanobacteria are able to maximize carbon balance during brief periods of favorable conditions due to rapid metabolic response (Figure 5). We propose that as with the cryptogams and some invertebrates (Convey, 1996), this ability to “live by the hour” is the key organismal trait for successful colonization of the arid polar biome by unicellular photoautotrophs.

**FIGURE 6:**
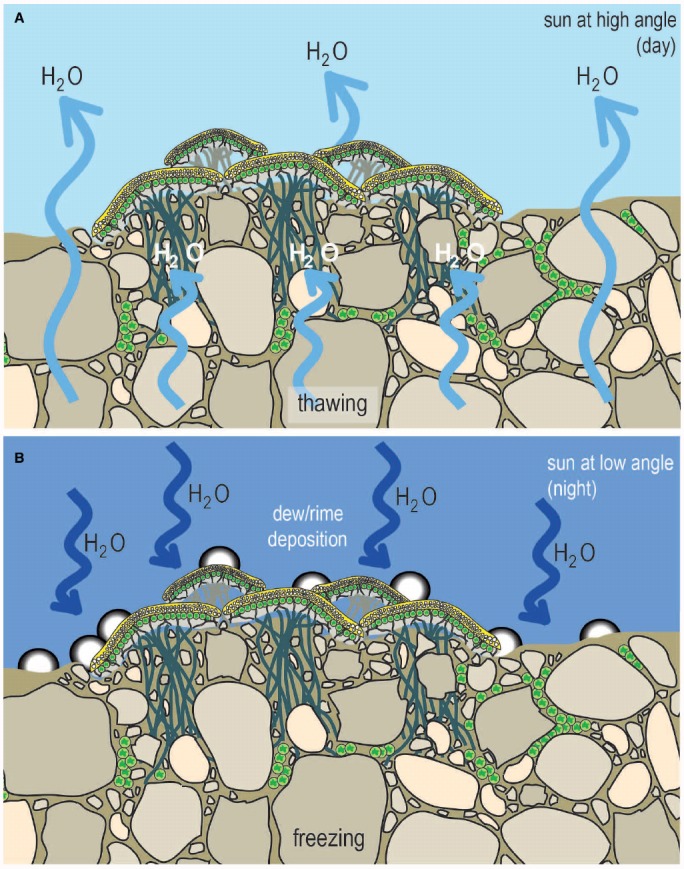
**Microclimate dynamics for cryptogamic photoautotrophs.** Biological soil crusts and endolithic colonization form extensive near-surface biological covers in polar regions that define the critical zone of biological activity and the dry limit for photoautotrophic colonization on Earth (Pointing and Belnap, 2012). They occur in regions where precipitation is insufficient to sustain higher plant life. Their source of moisture arises from the creation of a substrate-air thermal gradient that supports dew/rime formation (Büdel et al., 2008; Büdel and Colesie, 2014). During periods of high sun angle (daytime) thawing of permafrost and soil water releases water to the critical zone **(A)**, when the sun is at a low angle (night) the thermal differential between substrate and air results in dew formation **(B)**.

Polar lichens have been shown to allocate relatively high amounts of fixed carbon to survival (rather than growth and reproduction) compared to temperate lichens (Colesie et al., 2014). Similarly mosses have a relatively high metabolic demand relative to carbon fixation (Green and Lange, 1995). However, they can “afford” to grow slowly as there is limited competition for space and their poikilohydric nature allows persistence over multi-century timescales (Green and Lange, 1995). This is undoubtedly also due in part to the need for exposed plants to produce UV-protective compounds, which are widespread in bryophytes, lichens, biological soil crusts and cyanobacterial mats at hyporheic margins of lakes (Post, 1990; Vincent et al., 1993; Büdel et al., 1997; Bollard-Breen et al., 2014). Some microbial photoautotrophs have achieved the consummate feat of polar habitat preference by colonizing cryptic habitats within and beneath the surface of soil or rock (Chan et al., 2012; Pointing and Belnap, 2012; Wierzchos et al., 2012). The internal pore spaces of soils and weathered rocks such as sandstone and granite provide a stable environment protecting from wind scour and UV radiation (Pointing and Belnap, 2012). This microenvironment supports elevated temperatures compared to surrounding air due to solar gain from the substrate (Kappen and Friedmann, 1983; McKay and Friedmann, 1985). A major advantage also accrues from moisture gain due to dew/rime deposition that occurs as a result of thermal differentials between substrate and air (Büdel et al., 2008; Büdel and Colesie, 2014; Figure 6). This occurs both during vaporization of permafrost during warmer temperatures, and also due to dew/rime deposition at colder temperatures, and this may in part determine the depth of colonization.

## What Drives the Disparity Between Distribution of Arctic and Antarctic Photoautotrophs?

Temperature alone cannot explain the striking absence of vascular seed plants in the southern polar region nor can it explain low diversity of other photoautotrophs relative to the Arctic. Colonization of islands from continental land is, however, limited by isolation, island size and island age (MacArthur and Wilson, 1967; Whittaker et al., 2008). Antarctica not only remains substantially isolated in contrast to the Arctic, but temperatures suitable for substantial recolonisation by vascular plants have likely only been available since the Holocene warming (Figure 2) and so shorter time for plant colonization is also a constraining factor. In non-polar environments spore-dispersed photoautotrophs are as diverse on isolated islands as they are on the continent in contrast to seed plants which are considerably less diverse on islands (Patiño et al., 2014). This implies that seed plant diversity on isolated land is limited by dispersal, but cryptogam diversity is less constrained by this mechanism. In the Antarctic, for example, it has recently been demonstrated that the “cosmopolitan” moss *Bryum argenteum* colonized the continent on at least three separate occasions in the last 0.5 to 4 million years (Pisa et al., 2014).

Major barriers against dispersal to Antarctica likely exist even for cryptogams because prevailing air circulation patterns do not result in long-distance direct bi-polar transfer of propagules and there is a general absence of proximal non-polar refugia, such as alpine and geothermal regions, available for stepping stone dispersal (Fraser et al., 2014; Pointing et al., 2014). Of the 380 lichen species known from Antarctica some 50% are considered endemic (Ovstedal and Lewis Smith, 2001), while only 8–10% of the 1750 Arctic lichen species are considered to be endemic (Dahlberg and Bultmann, 2013). This is thought to be due to the relative proximity of non-polar land to the Arctic.

Microbial photoautotrophs are assumed to display a more cosmopolitan distribution due to allometric considerations and a relative lack of barriers to airborne dispersal (Pearce et al., 2009). For example the same cyanobacterial taxa as defined by rRNA genes can be found in Arctic, Antarctic and alpine lakes (Jungblut et al., 2010). Conversely some polar cyanobacteria occupying highly cryptic habitats (e.g., hypolithic cyanobacteria) have not experienced gene flow for extended time scales and these may be considered phylogenetically endemic (Bahl et al., 2011). Recently, combined molecular, ecological and morphological studies have indicated far greater microbial endemism than previously assumed (Vyverman et al., 2010). Whether this corresponds to functionally distinct ecotypes is unresolved, although the prevalence of such endemic taxa suggests that isolation is perhaps limiting in Antarctica, not only for seed plants, but also to some extent for unicellular photoautotrophs. This is supported by a recent study of aerosolised microorganisms in the McMurdo Dry Valleys of Antarctica, where the majority of airborne taxa were found to be of local origin and with evidence for environmental selection against non-polar taxa (Bottos et al., 2014).

Barriers to dispersal may not be the only challenge facing propagule dispersal to Antarctica. Spores may be arriving regularly but the extreme environment severely filters those that can survive because there is no climatic gradient available in close proximity to Antarctica for gradual adaptive selection. Additionally, *in situ* speciation of all taxa in Antarctica might be limited due to slow rates of evolution and speciation in cold and dry environments (Rohde, 1992; Goldie et al., 2010; Gillman and Wright, 2014), a factor less obvious in the Arctic due to lack of dispersal barriers. We therefore suggest dispersal limitation, along with a lack of evolutionary time with a suitable climate for speciation and possibly climate-limited *in situ* rates of evolution might in combination explain the disparity between Arctic-Antarctic photoautotroph richness.

## Threats and Conservation Priorities

Polar regions are the fastest warming on the planet (Overpeck et al., 1997; Serreze et al., 2000; Turner et al., 2005; Bromwich et al., 2013) and so their biota is critically at risk from climate change. The “greening” of polar regions in response to climate change is widely expected and has a high profile in public and political arenas. Worryingly, the development of more productive polar plant communities may actually reduce carbon reservoirs in soils due to greater decomposition rates (Hill et al., 2011), thus creating a positive feedback where carbon release leads to further climate warming.

Predicted changes in Arctic vegetation are pressing concerns for the Arctic Council, and a Red List of threatened Arctic plants has been prepared, but to date this is limited to vascular species (Gillespie et al., 2012). Plant biomass is increasing in some Arctic locations (Tape et al., 2006; Walker et al., 2006; Hudson and Henry, 2009), and invasive colonization by previously “non-Arctic” species is likely with further warming (Hobbie, 1996). In the Antarctic, and concurring with recent IPCC assessments, the detailed and regionally focussed “Antarctic Climate Change and the Environment” report (Turner et al., 2009) concluded that climate change would result in new habitats for colonization by both native and invading flora and fauna. Monitoring for invasives in the Antarctic is, however, currently based on remarkably little robust data, other than monitoring studies of the two indigenous flowering plants from a single and small west Antarctic Peninsula location in the Argentine Islands (Fowbert and Smith, 1994; Parnikoza et al., 2009), and ongoing work on Signy Island in the South Orkney Islands (P. Convey, personal communication). Therefore no wide-scale data are yet available against which to assess predictions. Peninsula and mainland Antarctic terrestrial ecosystems currently have very few established non-native species compared to the sub-Antarctic islands (Convey and Lebouvier, 2009; Hughes and Convey, 2012) or even compared to parts of the High Arctic such as the Svalbard archipelago (Coulson et al., 2014). A dilemma for scientists and policy makers is therefore how to determine the native/non-native status and natural versus anthropogenic dispersal mechanisms of a newly discovered polar species, since treaty terms require different and mutually exclusive management strategies depending on native/non-native status (Hughes and Convey, 2010).

A critical knowledge gap occurs for non-vascular plants. The Arctic Council’s biodiversity working group, the Conservation of Arctic Flora and Fauna (CAFF), recently identified that the lichens and bryophytes of the high Arctic may be especially prone to future change (Christensen et al., 2013). Furthermore, sub-Antarctic lichens have been identified as capable of invasive colonization in a warming Antarctic continent (Sancho et al., 2007). The metabolic plasticity and capacity for rapid growth among free living cyanobacteria and chlorophytes suggests rapid responses in microbial photoautotrophic biomass and productivity, along with extinction threats to endemic taxa, should also be considered in future change scenarios for the high polar biome.

## Concluding Remarks

This is the first comprehensive review of biogeography for all the major photoautotrophic phyla (vascular and non-vascular plants plus microbial photoautotrophs) in the high polar biome beyond the treeline. We highlight the dominance of non-vascular plants and microbial photoautotrophs in many high latitude regions. Microclimate emerges as the major driver of transitions between photoautotrophic phyla, whereas life cycle and stature in higher plants and poikilohydry in non-vascular plants are the key biotic traits that govern organismal response. We highlight that although vascular plants are considered in polar conservation frameworks, there are gaps with regard to recognizing the ecological importance of cryptogams and photoautotrophic microorganisms, and identifying appropriate responses to the threat from invasive species in a warming world.

### Conflict of Interest Statement

The Review Editor Jayne Belnap declares that, despite having collaborated with the authors Steve Brian Pointing and Burkhard Buedel, the review process was handled objectively. The authors declare that the research was conducted in the absence of any commercial or financial relationships that could be construed as a potential conflict of interest.
